# Electrocatalytic Properties of Pulse-Reverse Electrodeposited Nickel Phosphide for Hydrogen Evolution Reaction

**DOI:** 10.3389/fchem.2021.781838

**Published:** 2021-12-13

**Authors:** Woohyeon Jo, Dasol Jeong, Jaebum Jeong, Taegeon Kim, Seungyeon Han, Minkyu Son, Yangdo Kim, Yong Ho Park, Hyunsung Jung

**Affiliations:** ^1^ Nano Convergence Materials Center, Korea Institute of Ceramic Engineering & Technology (KICET), Jinju, South Korea; ^2^ Department of Materials Science and Engineering, Pusan National University, Busan, South Korea; ^3^ Departments of Materials and Chemical Engineering, Hanyang University, 55 Hanyangdaehak-ro, Sangnok-gu, Ansan, South Korea

**Keywords:** nickel phosphide, pulse-reverse electrodeposition, hydrogen evolution reaction, Ni precipitate, volmer-heyrovsky route

## Abstract

Nickel phosphide (Ni-P) films as a catalytic cathode for the hydrogen evolution reaction (HER) of a water splitting were fabricated by a pulse-reverse electrodeposition technique. The electrochemical behaviors for the electrodeposition of Ni-P were investigated by the characterization of peaks in a cyclic voltammogram. The composition of the electrodeposited Ni-P alloys was controlled by adjusting duty cycles of the pulse-reverse electrodeposition. The HER electrocatalytic properties of the Ni-P electrodeposits with an amorphous phase as a function of phosphorous contents existing in Ni-P were electrochemically characterized by the analysis of overpotentials, Tafel slopes, and electrochemical impedance spectrometry. Additionally, the elemental Ni-embedded crystalline Ni_3_P was prepared by an annealing process with the amorphous Ni_69_P_31_ electrodeposit with high contents of phosphorus. The crystalline structure with Ni inclusions in the matrix of Ni_3_P was formed by the precipitation of excess Ni. The electrocatalytic properties of crystalline Ni_3_P with elemental Ni inclusions were also investigated by electrochemical characterization.

## Introduction

Research on environmentally friendly renewable energy has been conducted to replace fossil fuels with limited reserves. Hydrogen energy (H_2_) with high gravimetric density has been researched as a candidate for an environmentally friendly sustainable energy source (Kapdan and Kargi, 2006; Nikolaidis and Poullikkas, 2017). Nowadays, hydrogen was dominantly produced by the steam reforming of natural gases with the emission of carbon dioxide as a by-product (Muradov and Veziroǧlu, 2005). Electrochemical water splitting operating in a sustainable manner, such as wind and solar power, has been applied to produce green hydrogen as a promising approach. Water splitting is a chemical reaction in which water is separated into hydrogen and oxygen by applying an electric current. The representative technologies of electrochemical water splitting can be categorized in alkaline water electrolysis (AEL), proton exchange membrane electrolysis (or polymer electrolyte membrane) (PEMEL), and solid oxide electrolysis (SOEL) (Brisse et al., 2008; Carmo et al., 2013; David et al., 2019; Shiva Kumar and Himabindu, 2019; Brauns and Turek, 2020). The water splitting reaction was typically performed in the corrosive media of an acidic or alkaline electrolyte. The fabrication of stable and efficient electrocatalysts with low overpotentials for both hydrogen evolution reaction (HER) and oxygen evolution reaction (OER) in water splitting is still a challenging issue. Pt for HER and Ir/Ru oxides as commercial electrocatalytic materials have been utilized. However, the high cost of noble materials is a key limiting factor for application to a large-scale system. Non-noble materials such as metal oxides, chalcogenides, sulfides, nitrides, carbides, and phosphides have been investigated to replace precious electrocatalytic materials (Brown et al., 1984; Raj and Vasu, 1990; Liu and Rodriguez, 2005; McKone et al., 2011; Merki and Hu, 2011; Vrubel and Hu, 2012; Jiang et al., 2014; Kucernak and Sundaram, 2014; Pu et al., 2014; Pan et al., 2015a; Pan et al., 2015b; Wang et al., 2015; Chen et al., 2018; Zhao et al., 2020). Among them, nickel phosphide (Ni-P) compounds have been attracting attention as one of the promising candidates for both HER and OER electrocatalysts. The Ni-P compounds typically exist in the crystalline phases of Ni_3_P, Ni_12_P_5_, Ni_2_P, and Ni_5_P_4_. The relatively higher positive charges of Ni^δ+^ and the stronger ensemble effect of phosphorous with increasing contents of phosphorous in Ni-P compounds have been reported to improve the catalytic activities especially for HER due to the easier desorption of H_2_ (Pan et al., 2015b; Menezes et al., 2017). The Ni-P electrocatalysts for water splitting have been fabricated by various approaches, such as colloidal synthesis, phosphorylation, physical vapor deposition, chemical vapor deposition, plasma spraying method, and electrodeposition (Wu and Duh, 2003; Mahalingam et al., 2007; Panneerselvam et al., 2008; Wu and Wu, 2013; Cai et al., 2015; Kornienko et al., 2017; Hasannaeimi and Mukherjee, 2019; Kim et al., 2019; Suryawanshi et al., 2020). The electrodeposition technique can be performed to deposit homogeneous Ni-P electrocatalysts in low temperature and atmospheric pressure without high-cost facilities. The contents of phosphorous in Ni-P electrodeposits have been controlled to enhance the electrocatalytic performance by adjusting electrochemical parameters such as the concentration of phosphorus acid, the applied voltage, the applied current, and deposition time (Bonino et al., 1997; Bai et al., 2003; Krolikowski et al., 2006; Mahalingam et al., 2007; Pu et al., 2014; Jiang et al., 2016; Hasannaeimi and Mukherjee, 2019). However, the phosphorous contents in Ni-P electrodeposits are difficult to increase with typical potentiostatic or galvanostatic deposition techniques, since the deposition was conducted by the indirect electrodeposition mechanism called induced co-deposition (Mahalingam et al., 2007; Hasannaeimi and Mukherjee, 2019). Compared to the potentiostatic/galvanostatic and typical pulse electrodeposition techniques with the limited phosphorous contents in Ni-P compounds, a pulse-reverse electrodeposition technique can be employed as the efficient approach to increase the contents of phosphorous in Ni-P compounds. The relative phosphorous contents in Ni-P electrocatalysts can be increased by the selective dissolution of Ni elements in an anodic reaction for the reverse pulse. Therefore, the composition of Ni-P electrocatalysts can be minutely controlled by tailoring a duty cycle of pulse-reverse electrodeposition. A few studies for Ni-P pulse-reverse electrodeposition have been reported (Wu and Wu, 2013; Wu et al., 2015; Kim et al., 2019).

In this paper, the electrochemical behavior of the Ni-P compound was analyzed by cyclic voltammetry. Ni-P compounds as a HER electrocatalyst for the water splitting were fabricated by utilizing a pulse-reverse electrodeposition technique. Compared to the typical Ni-P prepared by a potentiostatic deposition and a pulse electrodeposition, the composition of the amorphous Ni-P electrocatalysts was systematically controlled by controlling the duty cycle of applied pulses in the pulse-reverse electrodeposition. The electrocatalytic properties and the electrochemical behaviors of Ni-P compounds for HER of water splitting in acidic media were intensely investigated as a function of the tailored contents of phosphorous in Ni_x_P_y_ by polarization curves, Tafel plots, and Nyquist plots. Additionally, the electrodeposited Ni-P electrocatalysts with an amorphous phase were annealed. The electrocatalytic properties of the crystallized Ni-P compound were systematically analyzed.

## Materials and Methods

The Ni-P films were fabricated by pulse-reversed electrodeposition at 30°C using a typical three-electrode configuration with a platinum (Pt) mesh and a saturated calomel electrode (SCE) as the counter electrode and reference electrode, respectively. Fluorine-doped tin oxide glasses (∼7 Ω/sq., Sigma-Aldrich, St. Louis, MO, USA) with an area of 25 × 25 mm were employed as the working electrode. Fluorine-doped tin oxide (FTO) glass substrates were ultrasonically cleaned with deionized water (18 MΩ-cm) and a mixture of ethanol and acetone (v/v ratio 1:1) for 20 min and then dried at 80°C for 30 min under air. The electrolyte for the Ni-P electrodeposition consisted of 0.5 M nickel(Ⅱ) acetate tetrahydrate (Sigma-Aldrich), 0.29 M phosphorous acid (Daejung Chemical, Siheung, South Korea), 0.65 M phosphoric acid (Junsei Chemical, Tokyo, Japan), and 5 wt.% N-methylformamide (NMF, Sigma-Aldrich) with a pH of 3.4. The pulse-reverse electrodeposition was performed by the periodic application of a voltage of -0.85 V (vs. SCE) for the cathodic Ni-P co-deposition reaction and a reverse voltage of -0.2 V (vs. SCE) for the anodic Ni dissolution reaction. The composition of Ni-P electrodeposits was controlled with the varied duty cycles (duty cycle = $\frac{t_{cathodic}}{t_{cathodic}+t_{anodic}}\times 100$ ). The numbers of periodic cycles for the fixed film thickness of about 500 nm were determined by Faraday’s law. The crystalline Ni-P catalyst was prepared by an annealing process at 500 °C in Ar(g) for 1 h with a heating rate of 5°C/min.

Material characterization was carried out with an X-ray diffractometer (XRD, D8 Advance, Bruker, Billerica, MA, USA) with Cu-Kα radiation (*λ* = 1.5418 nm), field-emission scanning electron microscope (FE-SEM, JSM-6700, JEOL, Tokyo, Japan), transmission electron microscope (TEM, Titan Themis Z, FEI), and energy-dispersive x-ray spectroscope (EDS) equipped with TEM and SEM. Electrochemical analysis was performed with an electrochemical workstation (PMC-1000, AMETEK, Berwyn, PA, USA). The cyclic voltammetry was measured with the scan range of -1.0–1.0 V (vs. SCE) at the scan rate of 50 mV/s in the Ni-P electrolyte. Polarization curves, Tafel plots, and Nyquist plots for the analysis of HER electrocatalytic properties were measured in the acidic solution of 0.5 M H_2_SO_4_. The linear sweep voltammetry for polarization curves and Tafel plots was swept from 0 to -0.62 V (vs. RHE) at the scan rate of 5 mV/s. Electrochemical impedance spectrometry was performed by applying a potential at -325 mV with an amplitude of 10 mV in the frequency range of 10 kHz to 1 Hz. The electrochemical capacitance surface area (ECSA) was calculated by the double-layer capacitance (C_dl_) obtained from the cyclic voltammetry at scan rates from 10 to 800 mV/s in the non-Faraday region nearest to the HER-evolving potentials. Specific capacitance (C_s_) was assumed to be 40 μF/cm^2^ (Han et al., 2019). The accelerated degradation test (ADT) was performed by sweeping for 2,500 cycles at the scan rate of 100 mV/s with the potential range of 0 to -0.62 V (vs. RHE) in the acidic solution of 0.5 M H_2_SO_4_.

## Results and Discussion

**FIGURE 1:**
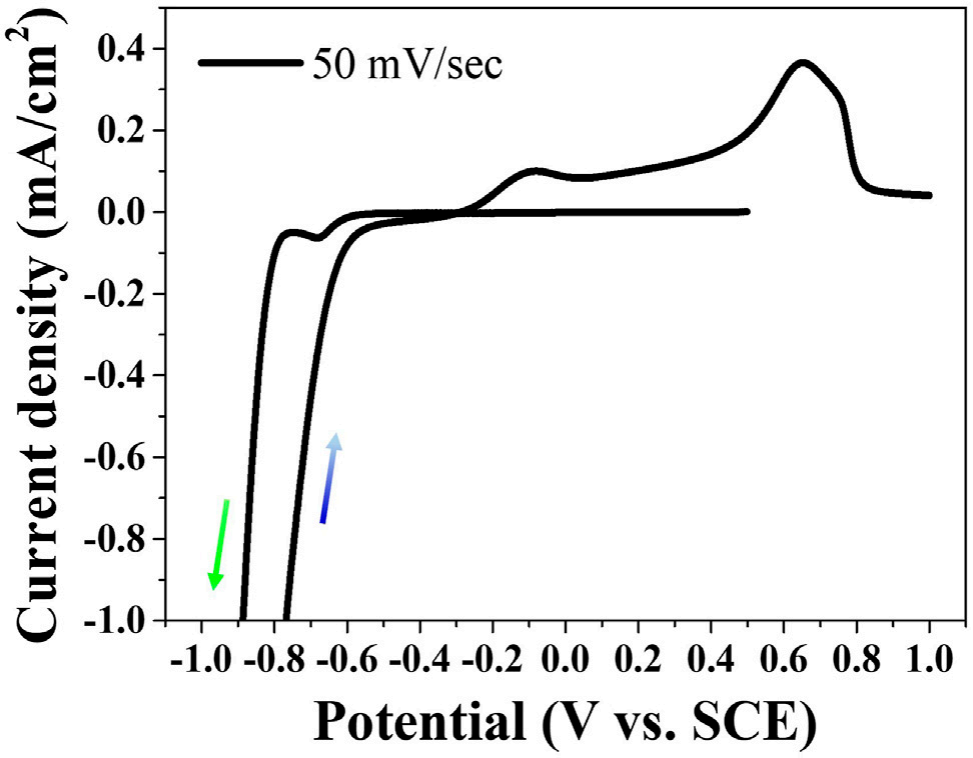
Cyclic voltammetry behavior for Ni-P electrodeposition.

Phosphorous in aqueous solutions cannot be electrodeposited alone due to the high cathodic reduction potential over HER. However, the metal phosphides including the phosphorous element can be electrodeposited by the process defined as the induced co-deposition. Figure 1 shows the cyclic voltammetry behavior swept at the scan rate of 50 mV/s on an FTO substrate in the aqueous electrolyte including Ni(OCOCH_3_)_2_·4H_2_O, H_3_PO_3_, H_3_PO_4_, and N-CH_3_NHCHO. The reduction peak at approximately -0.68 V (vs. SCE) in the cathodic sweep appeared indicating the electrodeposition of Ni-P alloy. Although the mechanism of Ni-P induced co-deposition is still unclear, the electrodeposition of Ni-P has been proposed by an indirect mechanism, as described in Eqs 1–3. The formation of Ni-P is achieved by the reaction of Ni^2+^ ions with PH_3_ as an intermediate (Mahalingam et al., 2007; Hasannaeimi and Mukherjee, 2019). Two peaks for oxidation reactions were observed in the anodic scan. The first peak at about -0.08 V (vs. SCE) and the second peak at about 0.65 V (vs. SCE) indicate the oxidation of Ni and the dissolution of Ni-P, respectively (Crousier et al., 1993).

$$6H^{+}+\;6e^{-}\;\to \;6H_{\mathrm{ads}}$$ (1)

$$H_{3}{\mathrm{PO}}_{3}+6H_{\mathrm{ads}}\to \;{\mathrm{PH}}_{3}\;+\;3H_{2}O$$ (2)

$$2{\mathrm{PH}}_{3}+\;3{\mathrm{Ni}}^{2+}\to \;3\mathrm{Ni}\;+\;2P\;+\;6H^{+}$$ (3)

**FIGURE 2:**
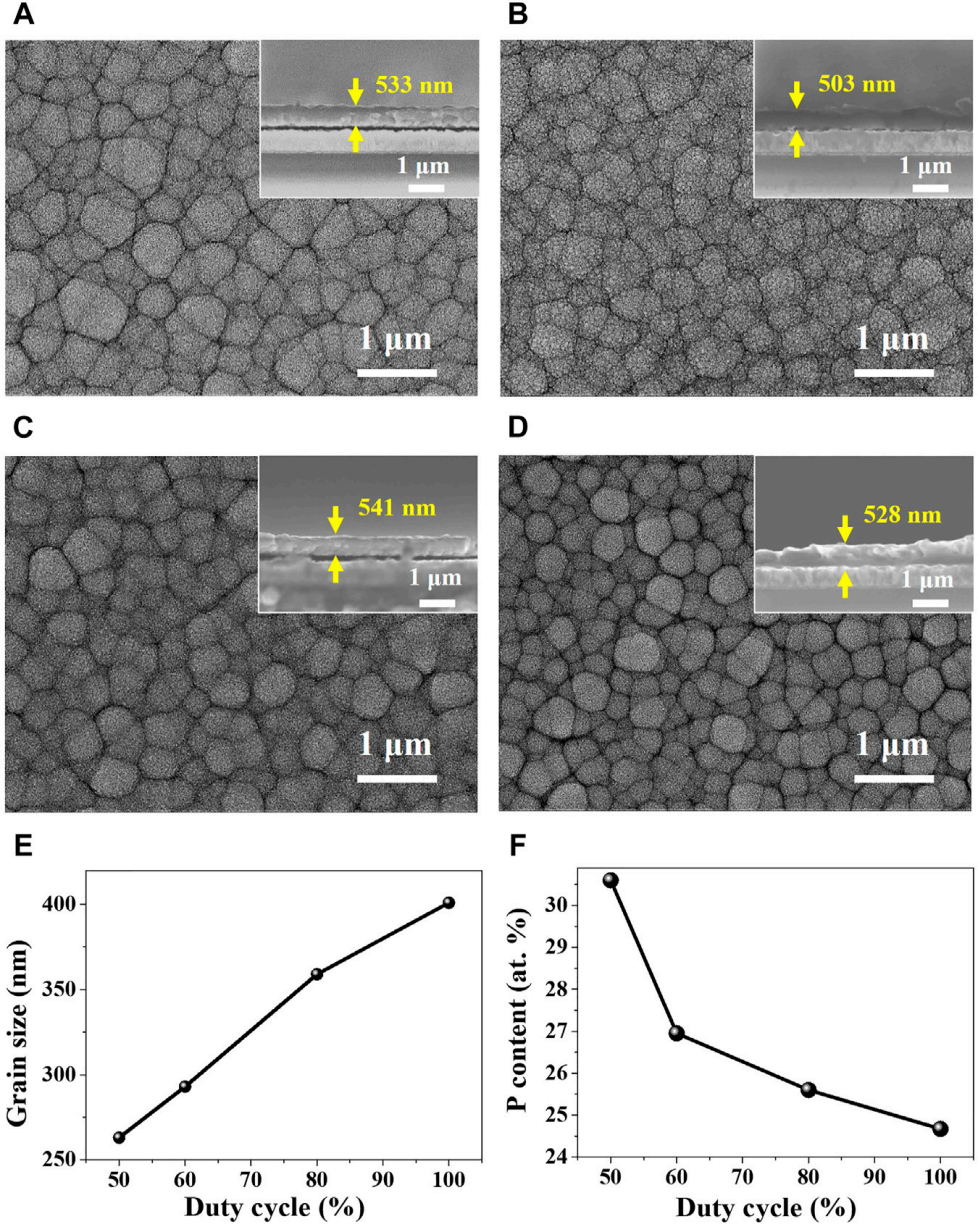
Surface morphologies of Ni-P pulse-reverse electrodeposited at tailored duty cycles: **(A)** 100% (DC), **(B)** 80%, **(C)** 60% and **(D)** 50% (insets: cross-sectional views), and **(E)** the average grain size and **(F)** the phosphorous contents of the Ni-P electrodeposits as a function of duty cycles.

Based on the cyclic voltammetry study for the electrochemical behavior of Ni-P, Ni-P electrodeposits with the tailored contents of phosphorous were prepared by pulse-reverse electrodeposition with the controlled duty cycles for the cathodic reaction of the Ni-P co-deposition and the anodic reaction of the dissolution of Ni. Figures 2A–D show the morphologies of the Ni-P compounds pulse-reverse electrodeposited at the tailored duty cycles of 50%, 60%, 80%, and 100% (DC), respectively. The thickness of Ni-P electrodeposits was controlled at about 527 ± 12.2 nm, as shown in insets of Figures 2A–D. The surface morphologies of Ni-P electrodeposits display the grained structures. Image analyses of the average grain size were accomplished by a linear intercept method. The average grain size of Ni-P electrodeposits decreased with the decrease in the applied duty cycle, as shown in Figure 2E. The increased ion diffusion on a cathode at the lower duty cycle with the longer anodic reaction time can induce the frequent adsorption of ionic species. Therefore, the decrease in the duty cycle of the pulse-reverse Ni-P electrodeposition can lead to the increase in the nucleation rate and the inhibition of the grain growth (Chandrasekar and Pushpavanam, 2008; Wahyudi et al., 2019). The average grain size of Ni-P electrodeposits decreased from 401 nm for the duty cycle of 100% to 263 nm for the duty cycle of 50%. Additionally, the EDS analysis of Ni-P electrodeposited as a function of the tailored duty cycles is described in the graph of Figure 2F. The phosphorous contents in the Ni-P electrodeposit increased with the decrease in the duty cycle. Ni-P electrodeposits at the duty cycles of 100%, 80%, 60%, and 50% indicated the phosphorous contents of 24.7, 25.6, 27.0, and 30.6 at.%, respectively. The phosphorous content of 30.6% in Ni-P pulse-reverse electrodeposited at the duty cycle of 50% showed the relatively high 30.6 at.%, compared to the phosphorous contents in the Ni-P compounds prepared with conventional DC electrodeposition methods ranging from 12 to 28 at.% (Bonino et al., 1997; Bai et al., 2003; Krolikowski et al., 2006; Mahalingam et al., 2007; Hasannaeimi and Mukherjee, 2019). The increase in phosphorous contents of Ni-P prepared at the lower duty cycle of the pulse-reverse electrodeposition can be attributed to the increased Ni dissolution reaction due to the longer anodic reaction time. The dissolution of Ni in the electrodeposited Ni-P compounds can increase the relative content of phosphorous. Furthermore, the indirect phosphorous co-deposition with Ni requires the indispensable presence of nascent hydrogen to produce PH_3_, as described in Eqs 1–3. The increased contents of phosphorous at the lower duty cycle might be attributed to the increased H^+^ ions adsorbed on a cathode required for the PH_3_ formation, because the longer anodic reaction time can effectively remove the reduced species such as H_2_ from the cathode surface (Hasannaeimi and Mukherjee, 2019).

**FIGURE 3:**
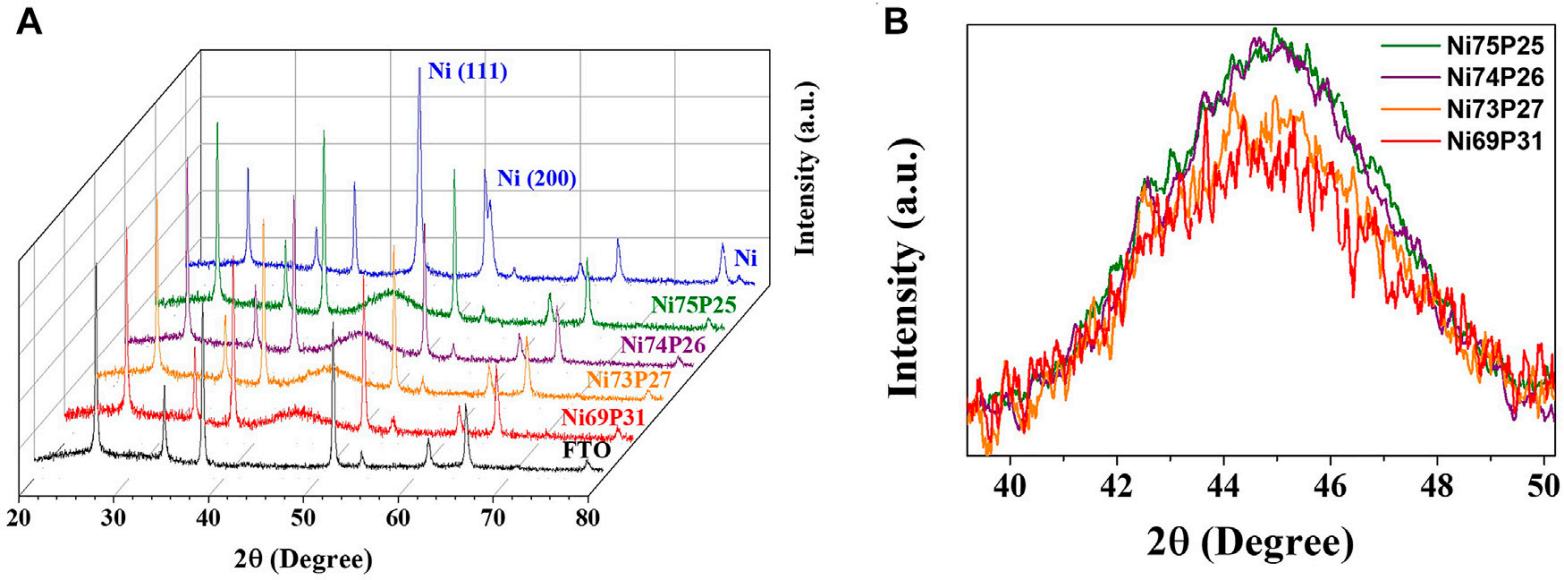
**(A)** XRD patterns of the pulse-reversed electrodeposited Ni-P with tailored composition and **(B)** the broad peaks around 44° with amorphous phase as a function of phosphorous contents.

Figure 3 shows the XRD spectra of the pulse-reverse electrodeposited Ni-P at the tailored duty cycles. The well-developed peaks in the diffraction patterns indicate the crystal planes of SnO_2_ in FTO substrates. The diffraction peaks were defined as the (110), (101), (200), (211), (310), and (301) planes of SnO_2_ (JCPDS, no. 46-1088). The diffraction patterns of the Ni-P electrodeposits were observed as a broad peak at about 44 for the (111) plane of Ni (JCPDS, no. 87-0712). The induced co-deposited phosphorous atoms with Ni for the Ni-P electrodeposition were incorporated into the Ni lattices. Compared to the XRD patterns of the polycrystalline Ni electrodeposit with well-developed peaks, the Ni-P electrodeposits showed the amorphous phase indicating the broad peaks at about 44°. As the contents of phosphorous in Ni-P electrodeposits increase, the crystallinity of Ni-P becomes poor. Additionally, the application of a pulse to enable interference in the continuous crystal growth can aggravate the short-range ordered crystallinity. Therefore, the Ni-P electrodeposits with the induced co-deposition mechanism typically exhibited the poor crystallinity or amorphous phase. Furthermore, the increase in phosphorous contents in Ni-P with decrease in duty cycles intuitively showed the gradual diminution of the intensity for the broad amorphous peak.

**FIGURE 4:**
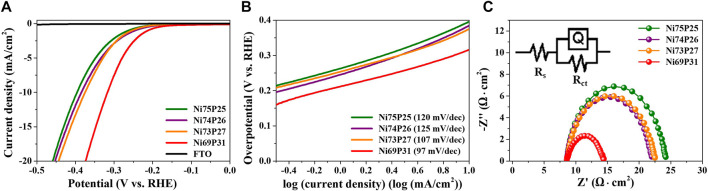
Electrocatalytic properties of the amorphous Ni-P electrodeposits as a function of contents of phosphorous: **(A)** polarization curves, **(B)** Tafel plots, and **(C)** Nyquist plots.

The electrocatalytic properties of amorphous Ni-P electrodeposits with tailored compositions were characterized as shown in Figure 4. Polarization curves were measured to characterize the overpotential for HER of amorphous Ni-P compounds in the acidic electrolyte of 0.5 M H_2_SO_4_ (Figure 4A). The electrocatalytic activity of a bare FTO substrate without Ni-P electrodeposits for HER was not observed. The overpotential of the commercial Pt/C for HER has been reported to be approximately -14 mV at -10 mA/cm^2^ (Kim et al., 2019). The overpotentials of Ni_69_P_31_, Ni_73_P_27_, Ni_74_P_26_, and Ni_75_P_25_ with the controlled compositions indicated -317, -375, -385, and -396 mV at -10 mA/cm^2^, respectively. The overpotential of amorphous Ni-P electrodeposits for HER was gradually reduced with the increase in the phosphorous contents. The enhanced electrocatalytic properties for HER might be attributed to the lower desorption energy of H_2_ due to the reduced negative charge on the surface of phosphorous to trap protons with increasing contents of phosphorous in Ni-P electrodeposits, as well as to the decreased active sites of Ni to reduce the H_2_ desorption energy (ensemble effect) (Pan et al., 2015b; Menezes et al., 2017). The kinetics of Ni-P electrodeposits for HER as a function of composition was investigated by Tafel analysis, as shown in Figure 4B. The Tafel slopes of Ni_69_P_31_, Ni_73_P_27_, Ni_74_P_26_, and Ni_75_P_25_ decreased with the increase in the contents of phosphorous, indicating 97, 107, 125, and 120 mV/dec, respectively. The mechanism for HER in acidic solutions can be described with three processes of the formation step of H_ads_ *via* the adsorption of H^+^ (Volmer reaction), the desorption step (Heyrovsky reaction), and the combination step (Tafel reaction), as described in Eqs 4–6.(Zeng and Li, 2015). The HER in acidic solutions can be determined by two successive steps of the Volmer–Tafel route or/and the Volmer–Heyrovsky route. The Tafel slopes of Volmer reaction, Heyrovsky reaction, and Tafel reaction as the rate-determining step are 118, 39, and 29.5 mV/dec (Zeng and Li, 2015). The Tafel slope of the Pt electrocatalyst for HER has been reported to be about 30 mV/dec indicating a Volmer–Tafel route with the Tafel reaction as a rate-determining step following the fast initial Volmer reaction. The HER process of Ni-P electrocatalysts has been typically described as Volmer–Heyrovsky route with the Tafel slopes of about 40–75 mV/dec (Pu et al., 2014; Pan et al., 2015b; Zhao et al., 2020). The Tafel slopes of the amorphous Ni-P electrodeposits indicated that the rate-determining step is the Volmer reaction in the mixed kinetic mechanism. The reduction in Tafel slopes with the increase in the contents of phosphorous implied the faster adsorption of intermediate hydrogen atoms on the surface of Ni-P catalysts. Electrochemical impedance spectrometry as a further kinetic study for HER was analyzed as a function of the composition of Ni-P electrodeposits. The charge transfer resistance of the interface between the Ni-P catalyst and electrolyte was drastically reduced in Ni_69_P_31_ with the highest contents of phosphorous. The enhanced charge transfer of the Ni_69_P_31_ electrocatalyst was in agreement with the results of the analyses of overpotentials and Tafel plots (Figure 4C).

$$H^{+}+e^{-}\;\to H_{\mathrm{ads}}\left(\mathrm{Volmer}\;reaction\right)$$ (4)

$$H_{\mathrm{ads}}+H^{+}+e^{-}\;\to H_{2\left(g\right)}\;\left(\mathrm{Heyrovsky}\;reaction\right)$$ (5)

$$H_{\mathrm{ads}}+H_{\mathrm{ads}}\;\to H_{2\left(g\right)}\left(\mathrm{Tafel}\;reaction\right)$$ (6)

**FIGURE 5:**
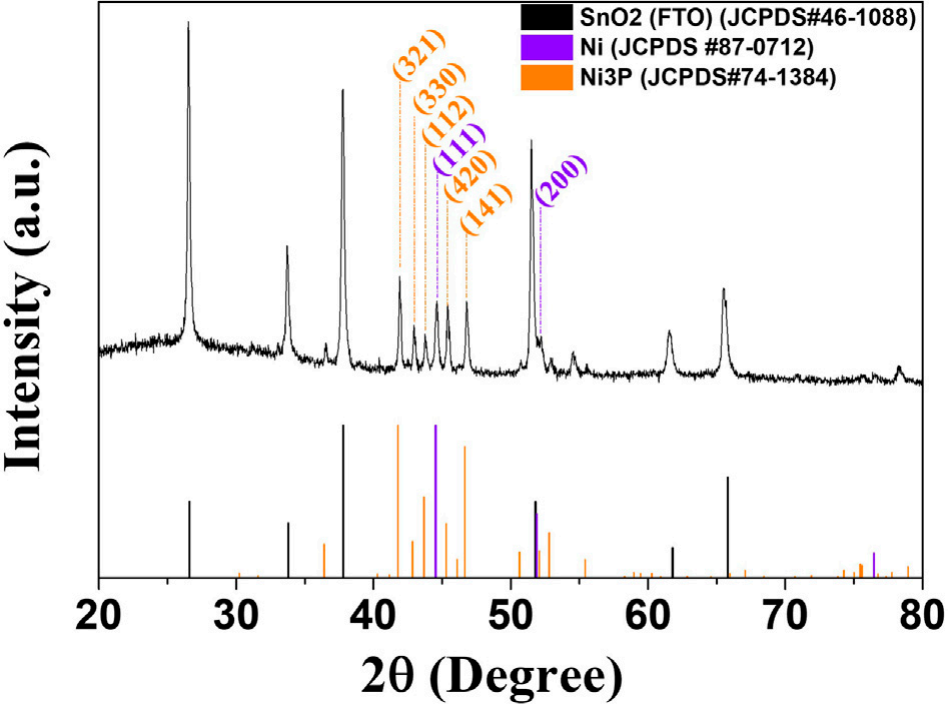
XRD patterns of the annealed Ni_69_P_31_ electrodeposit.

The Ni-P electrodeposits had amorphous phases as shown in Figure 3. Lowering of the duty cycles in the pulse-reverse electrodeposition induced Ni-P electrodeposits to become further amorphous, aggravating the short-range ordered crystallinity due to the brief periodic deposition time and increase in phosphorous contents. The amorphous Ni_69_P_31_ electrodeposited at the duty cycle of 50% was annealed at 500°C in Ar 7(g) for 1 h. Figure 5 shows the XRD patterns of the annealed Ni_69_P_31_ electrodeposit. Compared to the XRD pattern of the bare Ni_69_P_31_ electrodeposit, the diffraction patterns of annealed Ni_69_P_31_ shows the well-developed peaks, indicating the crystallization of amorphous Ni_69_P_31_. The diffraction peaks were defined as the (301), (321), (330), (112), (420), (141), and (321) planes of Ni_3_P (JCPDS, no. 74-1384) and the (111) and (220) planes of Ni (JCPDS, no. 87-0712). As described in XRD analysis, the amorphous Ni_69_P_31_ electrodeposit was transformed into two crystal structures of Ni_3_P and Ni by the annealing process.

**FIGURE 6:**
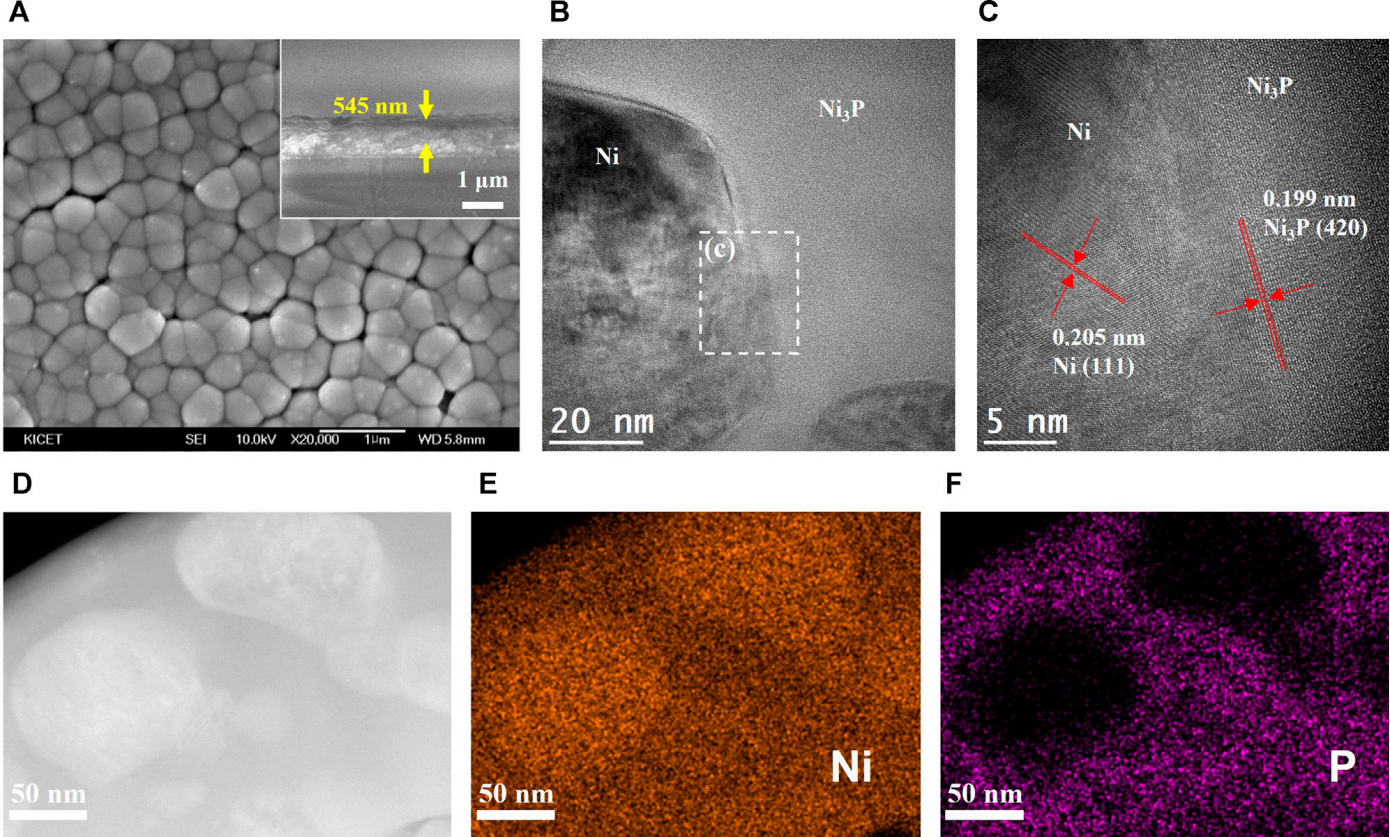
Structural characterization of the crystalline Ni_3_P with elemental Ni inclusions prepared by annealing of amorphous Ni_69_P_31_ electrodeposit: **(A)** FE-SEM image (SEM scale bar and thickness display), **(B)** bright-field TEM image, **(C)** HRTEM image, **(D)** STEM image, and **(E, F)** EDS elemental mapping for Ni and phosphorous.

SEM and TEM analyses of the annealed Ni_69_P_31_ electrodeposit was conducted as shown in Figure 6. The surface morphology of the crystallized Ni_69_P_31_ electrodeposit was similar to the surface of the as-deposited amorphous Ni_69_P_31_ with the grain boundaries, as shown in Figure 6A. Corresponding with the XRD analysis, TEM images of the annealed Ni_69_P_31_ electrodeposit clearly showed the interface between the Ni precipitates and Ni_3_P with well-developed lattice structures. The HRTEM image revealed a crystalline structure for Ni precipitates with a lattice spacing of 0.205 nm and for Ni_3_P with a lattice spacing of 0.199 nm, corresponding to the (111) planes of Ni (JCPDS, no. 87-0712) and the (420) planes of Ni_3_P (JCPDS, no. 74-1384). The elemental precipitation of Ni in the matrix of Ni_3_P can be obviously confirmed from STEM and EDS elemental mapping analysis, indicating the compositional distribution of Ni_3_P/Ni. The excess elemental Ni over Ni_3_P with the stoichiometric composition in Ni_69_P_31_ might be precipitated for the annealing process.

**FIGURE 7:**
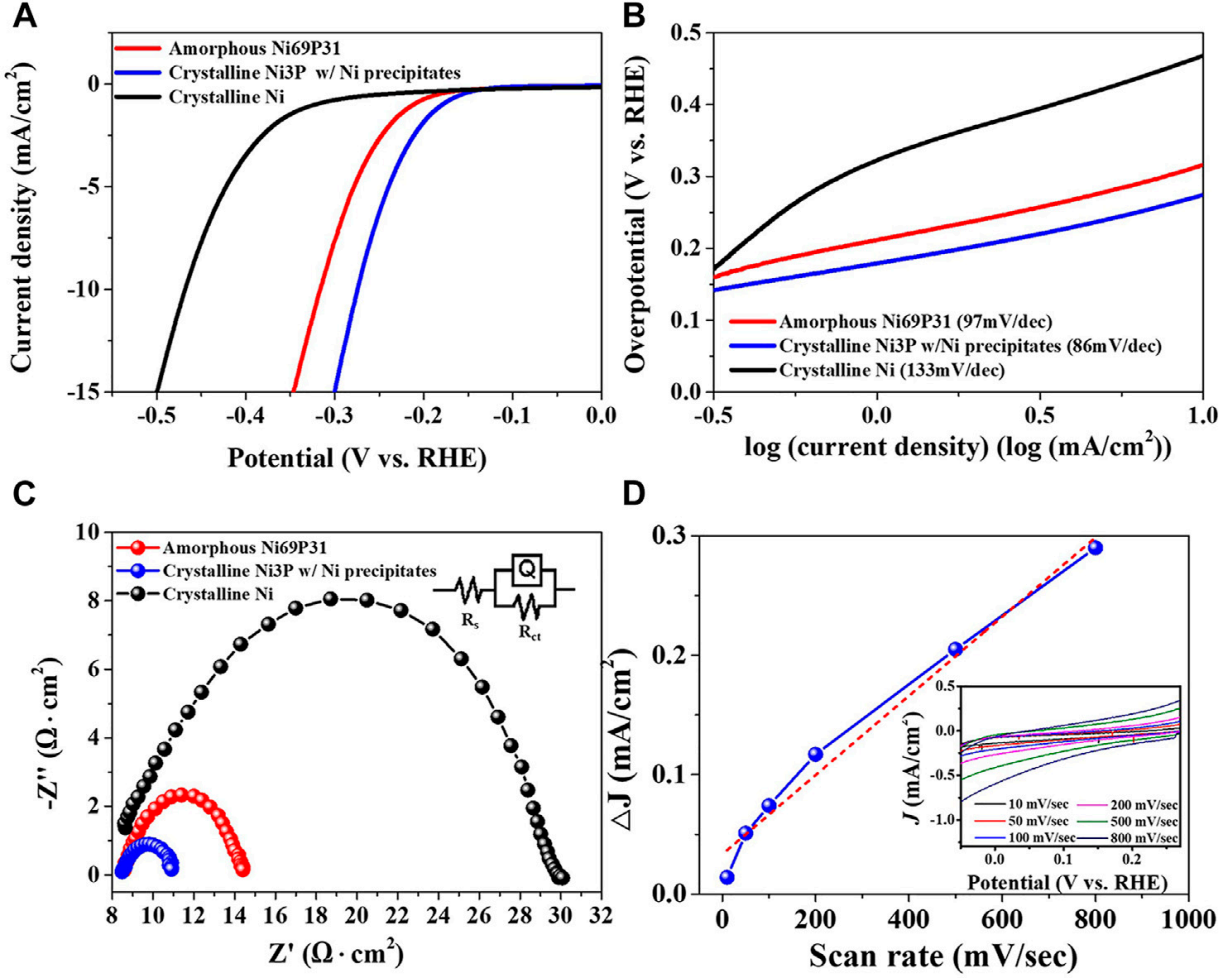
Electrocatalytic properties of the crystalline Ni_3_P with elemental Ni inclusions prepared by annealing of amorphous Ni_69_P_31_ electrodeposit: **(A)** polarization curves, **(B)** Tafel plots, **(C)** Nyquist plots, and **(D)** ECSA estimation based on the capacitive current at 250 mV (vs. RHE) (inset: cyclic voltammograms crystalline Ni_3_P with elemental Ni inclusions as a function of scan rate).

The electrocatalytic properties of crystalline Ni_3_P with elemental Ni inclusions were characterized as shown in Figure 7. The crystalline Ni_3_P with elemental Ni inclusions indicated the lower overpotential of -275 mV at -10 mA/cm^2^ for HER, compared to the HER overpotential of -317 mV in the amorphous Ni_69_P_31_ and the overpotential of -468 mV in the crystalline Ni film. The Tafel slope of the crystalline Ni_3_P with elemental Ni inclusions indicated 86 mV/dec which is smaller than the Tafel slope of 97 mV/dec and 133 mV/dec in the amorphous Ni_69_P_31_ and crystalline Ni film, respectively. Based on the Tafel slope of 86 mV/dec in the crystalline Ni_3_P with elemental Ni inclusions, the rate-determining step is still predicted to be the Volmer reaction in the Volmer–Heyrovsky route. However, the adsorption energy of intermediate hydrogen atoms on the surface of crystalline Ni_3_P with elemental Ni inclusions can be anticipated to be reduced, compared to the adsorption energy in the amorphous Ni_69_P_31_. The analysis of electrochemical impedance spectrometry showed the reduced charge transfer resistance of the interface between elemental Ni-embedded crystalline Ni_3_P and electrolyte. The electrocatalytic performance of Ni-P compounds for HER can be improved by the balanced control of the Volmer reaction for the adsorption of protons and the Heyrovsky reaction for the desorption of H_2_, considering the exposure of active sites with low energy barrier. The improved overpotential, Tafel slope, and charge transfer in the crystalline Ni_3_P with elemental Ni inclusions might be attributed to the lower desorption energy of hydrogen for Heyrovsky reaction and the lower adsorption energy for Volmer reaction. The energy for the H_2_ desorption (Heyrovsky reaction) in the crystalline Ni_3_P with Ni inclusions prepared from amorphous Ni_69_P_31_ with the highest phosphorous content was decreased by the enhanced ensemble effect. Moreover, the strong hydride formation in the Ni hollow sites of the crystallized Ni_3_P with Ni inclusions reduced the adsorption energy for proton adsorption reaction (Volmer reaction) called the ligand effect (Liu and Rodriguez, 2005; Chen et al., 2017). Additionally, the ECSA of the crystalline Ni_3_P with elemental Ni inclusions was characterized with the double-layer capacitance of the catalytic surface (McCrory et al., 2013). The double-layer capacitance (C_dl_) indicated 331.48 μF/cm^2^, which was calculated with the non-Faradaic double-layer charging current from the cyclic voltammograms at the varied scan rates. The calculated ECSA of the crystalline Ni_3_P with elemental Ni inclusions was 8.29 cm^2^ corresponding to the ECSA of typical electrodeposited Ni film with about 8.13 cm^2^ (Zhou et al., 2017).

**FIGURE 8:**
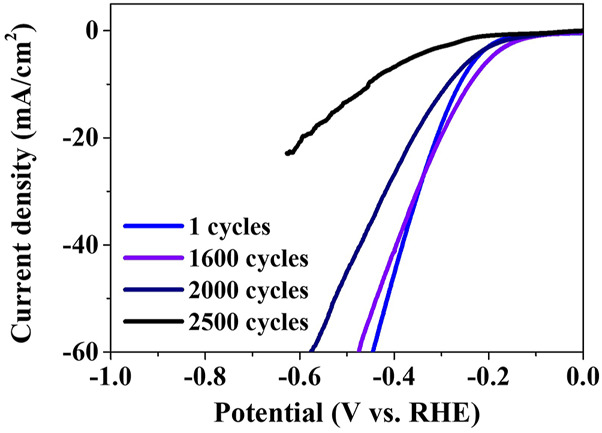
ADT of the crystalline Ni_3_P with elemental Ni inclusions up to 2,500 cycle.

The ADT was performed to evaluate the stability of the crystalline Ni_3_P with elemental Ni inclusions for the HER electrocatalyst, as shown in Figure 8. The ADT polarization curves showed the degradation of the electrocatalytic properties with the increase in the number of cycles. The cycle-dependent overpotentials in the current density of 10 mA/cm^2^ indicated -262 mV at 1 cycle, -241 mV at 1,600 cycles, -287 mV at 2,000 cycles, and -455 mV at 2,500 cycles, respectively. The polarization curve of 1,600 cycles slightly deviated with the overpotential of -241 mV, compared to the initial curve of 1 cycle with the overpotential of -262 mV. The severe deviation of the polarization curve with the overpotential of -455 mV after 2,500 cycles indicated the significant loss of the electrocatalytic properties.

## Conclusion

The electrochemical behaviors for the Ni-P electrodeposition were characterized by cyclic voltammetry, indicating the Ni-P co-deposition in cathodic sweep, the oxidation of Ni, and the dissolution of Ni-P in anodic sweep. The pulse-reverse electrodeposition including the Ni-P co-deposition and the dissolution of Ni was utilized to deposit Ni-P films with tailored compositions. Ni_69_P_31_, Ni_73_P_27_, Ni_74_P_26_, and Ni_75_P_25_ electrodeposits with the amorphous phase were prepared by adjusting the duty cycles of pulse-reverse electrodeposition. The overpotentials at -10 mA/cm^2^ and Tafel slopes of the amorphous Ni-P electrodeposits for HER were reduced from -396 and 120 mV/dec to -317 and 97 mV/dec, as the contents of phosphorous in Ni-P increased from Ni_75_P_25_ to Ni_69_P_31_. The analysis of electrochemical impedance spectrometry also showed the reduction of charge transfer resistance of the interface between Ni-P and electrolyte with increase in the contents of phosphorous. The improvement of electrocatalytic properties in amorphous Ni-P electrodeposits with increase in the contents of phosphorous can be achieved by the reduction of the H_2_ desorption energy. Additionally, crystalline elemental Ni-embedded Ni_3_P were prepared by the annealing of the amorphous Ni_69_P_31_ electrodeposit. The electrocatalytic properties of the crystalline Ni_3_P with elemental Ni inclusions for HER indicated the lower overpotential of -275 mV at -10 mA/cm^2^, the gentler Tafel slope of 86 mV/dec, and the reduced charge transfer resistance, compared to the properties of the amorphous Ni_69_P_31_. Compared to the HER catalytic properties in the amorphous Ni_69_P_31_, the enhanced electrocatalytic properties in the crystalline Ni_3_P with Ni inclusions might be attributed to the lower desorption energy of hydrogen for Heyrovsky reaction and the lower adsorption energy for Volmer reaction. The improved hydrogen desorption and proton adsorption processes for HER catalytic properties can be described by an ensemble effect and a ligand effect, respectively. The crystalline Ni_3_P with elemental Ni inclusions demonstrated the continuous stability for HER catalytic properties up to 1,600 cycles in acidic solution.

## Data Availability

The original contributions presented in the study are included in the article/Supplementary Material; further inquiries can be directed to the corresponding author.
